# Assessing what matters most to patients with or at risk for Alzheimer’s and care partners: a qualitative study evaluating symptoms, impacts, and outcomes

**DOI:** 10.1186/s13195-020-00659-6

**Published:** 2020-07-30

**Authors:** Dana B. DiBenedetti, Christina Slota, Samantha L. Wronski, George Vradenburg, Meryl Comer, Leigh F. Callahan, John Winfield, Ivana Rubino, Holly B. Krasa, Ann Hartry, Dan Wieberg, Ian N. Kremer, Debra Lappin, Allison D. Martin, Terry Frangiosa, Virginia Biggar, Brett Hauber

**Affiliations:** 1 RTI Health Solutions, Research Triangle Park, NC USA; 2 UsAgainstAlzheimer’s, Washington, DC USA; 3 University of North Carolina, Chapel Hill, NC USA; 4 Biogen, Inc., Cambridge, MA USA; 5 Blue Persimmon Group LLC, Washington, DC USA; 6 Lundbeck LLC, Deerfield, IL USA; 7 Home Instead, Inc., Omaha, NE USA; 8 LEAD Coalition (Leaders Engaged on Alzheimer’s Disease), Washington, DC USA; 9 Faegre Baker Daniels Consulting, Washington, DC USA

**Keywords:** Alzheimer’s disease, Caregiver, Outcome, Patient, Qualitative

## Abstract

**Background:**

The What Matters Most (WMM) study was initiated to evaluate symptoms, AD-related impacts, treatment-related needs, preferences, and priorities among individuals with or at risk for Alzheimer’s disease (AD) and their care partners. The objective of this qualitative study phase was to identify a comprehensive set of concepts of interest that are meaningful to individuals across the AD continuum.

**Methods:**

Interviews were conducted with 60 clinically referred individuals and care partners across 5 AD stages (*n* = 12 each): group 1 (non-clinically impaired individuals with AD pathology), group 2 (individuals with mild cognitive impairment and AD pathology), group 3 (individuals with mild AD), group 4 (individuals with moderate AD and their care partners), and group 5 (care partners of individuals with severe AD). Interviews were conducted by experienced interviewers, audio-recorded, and transcribed. Dominant trends were identified in each interview and compared across subsequent interviews to generate themes or patterns in descriptions of AD symptoms, impacts, and desired treatment outcomes.

**Results:**

All participants endorsed current issues related to memory; nearly all participants (*n* = 55; 92%) across the five groups endorsed symptoms related to communication and language. Groups 1–3 reported an impact on mood/emotions (*n* = 23; 64%) and a decrease in social activities or outgoingness (*n* = 17; 47%). Current and future concerns reported by the overall sample included memory (*n* = 48; 80%), dependence (*n* = 40; 67%), and “other” concerns (*n* = 33; 55.0%) (e.g., uncertainty about the future, burdening others). The most desired AD treatment outcomes were improvement or restoration of memory (*n* = 40; 67%) and stopping AD progression (*n* = 35; 58.3%). Group-level differences were observed in the symptoms, impacts, and desired treatment outcomes among patients and care partners across the AD continuum.

**Conclusions:**

Cognitive functioning issues—particularly in memory and communication—are present even in preclinical and early-stage AD, including among those without a formal AD diagnosis. While the impacts of AD vary across the disease-severity spectrum, improved memory and disease modification were treatment outcomes considered most important to participants across all 5 AD stages. Neuropsychological assessments traditionally used in AD clinical trials may not evaluate the often-subtle concepts that are important to patients and care partners. Results from this study will inform the second phase of the WMM project—a quantitative study to elicit the relative importance of these concepts of interest to people at risk for and living with AD and their care partners.

## Background

The What Matters Most (WMM) study, sponsored by the Alzheimer’s Disease Patient and Caregiver Engagement (AD PACE) consortium, is a two-part study designed to better understand and assess treatment-related needs (i.e., what matters) as well as treatment preferences and priorities (i.e., what matters most) among individuals with or at risk for Alzheimer’s disease (AD) and their care partners. Specifically, the goal of this research is to assess and better understand the AD symptoms, impacts, and treatment-related outcomes that matter to individuals with or at risk for AD and their care partners, as well as their treatment preferences and priorities. Such an in-depth understanding of the impact of AD on patients and care partners across the continuum of the disease is much needed to inform the development of AD treatments and programs, regulatory review of new drugs to treat AD, and health technology assessment and reimbursement decisions for new drugs and services to treat and manage AD.

Prior research on the conceptual relevance of neuropsychological assessments in AD has shown that existing measures may not capture the concepts that are most important to individuals with mild or mild-moderate AD, particularly the emotional and psychological impacts of the disease [5]. However, to our knowledge, no study yet has aimed to characterize the experiences and treatment priorities of patients and care partners across the AD severity spectrum, and the current study is unique in exploring these dimensions of the experiences of individuals affected by AD. This research is designed to be an initial step in developing a platform for continued information gathering, which may ultimately provide the necessary building blocks to support evaluations of the clinical meaningfulness of endpoints in clinical trials and observational studies as well as the development of novel patient-centered endpoints, patient-experience data, and core outcome sets. The intent of this research is to capture a snapshot of patient and care partner experiences, needs, preferences, and priorities, which are central to defining clinically meaningful treatment outcomes across the continuum of AD [4, 9, 12].

The WMM study is being conducted in two phases. The objective of phase 1, reported here, was to elicit the potential treatment-related outcomes that matter to people with or at risk for AD and their care partners. Phase 2 of the study will be a quantitative phase to estimate how much each potential treatment-related outcome matters and which potential treatment-related outcomes matter most.

## Methods

### Study population

In-depth interviews were conducted with 60 clinically referred individuals and care partners across a continuum of 5 AD stages (*n* = 12 each). The sample size was chosen to enable the identification of patterns in the emergent concepts identified from the interview data while supporting a detailed qualitative analysis [11]. Although evidence-based approaches to determining sample sizes for qualitative studies have not been defined [10], the size of the overall sample and AD groups was considered sufficient to accomplish the study objectives.

Eligibility for the study was determined based on prespecified inclusion criteria. Specifically, group 1 included individuals with unimpaired cognition but with evidence of AD pathology, group 2 included individuals with mild cognitive impairment (MCI) and evidence of AD pathology, and group 3 included individuals with a diagnosis of mild AD. For groups 4 and 5, informal care partners (i.e., unpaid, nonprofessional caregivers) were also involved in the interviews [1, 3]. Specifically, group 4 interviews included individuals with a diagnosis of moderate AD and their care partners (12 dyads interviewed together), whereas group 5 included care partners of individuals with a diagnosis of severe AD, who were interviewed independently (without their care recipients). Evidence of AD pathology in group 1 and group 2 was defined as positive findings of amyloid positron emission tomography scan or cerebrospinal fluid lumbar puncture within in the past 6 months. For participants in groups 3, 4, and 5, stage of disease was determined by the participant’s referring physician. Individuals with history of any other type of dementia, traumatic brain injury, cerebral vascular accident/stroke, or any mental or other medical condition that the patient’s physician felt would interfere with the patient’s ability to engage in an interview were not eligible for the study.

### Interview methods

Participants were recruited through Global Market Research Group, a specialty recruiting firm, and Raleigh Neurology Associates, a neurology clinic based in Raleigh, North Carolina. All patients and care partners were referred directly from clinical sites in five locations: Chicago, Illinois; St. Louis, Missouri; St. Paul, Minnesota; New Orleans, Louisiana; and Raleigh, North Carolina.

Participants in groups 1, 2, and 3 were interviewed directly and reported their current symptoms, the impact of symptoms on them, and their desired treatment outcomes. Patient and care partner participants in group 4 were interviewed as a dyad. When able, patient participants were asked to self-report on their symptoms, impacts, and desired treatment outcomes. In group 5, care partners of participants with severe AD were interviewed. Care partners in both groups 4 and 5 reported on symptoms they observed in their care recipients but reported the impacts of the care recipient’s symptoms on them as care partners and what they themselves desired in treatment outcomes.

All interviews lasted approximately 60 min and followed semi-structured interview guides (patient and care partner guides). The interview guides were used to ensure that data were collected in a systematic and consistent way and that the interview objectives were met, while also encouraging spontaneity of responses and a conversational tone throughout the interviews.

Content for the AD participant and care partner interview guides was selected from a number of resources. Specifically, concepts and domains included in the Alzheimer’s Disease Assessment Scale—Cognitive (ADAS-Cog) [6–8] and information from the American Psychiatric Association’s *Diagnostic and Statistical Manual of Mental Disorders*, *Fifth Edition* [2], informed development of the interview guides. Concepts to explore in the interviews were also identified by reviewing published literature, the US Food and Drug Administration (FDA) Early Alzheimer’s Disease draft guidance document [3], and data on file materials from AD PACE Industry sponsors (e.g., qualitative reports, preference studies). In addition, feedback from the AD PACE Executive Steering Committee, which includes both patient and care partner representatives, was incorporated into the interview guides. Minor revisions to the interview guides were made after the first 13 interviews (composed of participants representing each of groups 2–5). The revised patient and care partner interview guides were used for the remaining interviews.

All interviews were conducted in person by experienced qualitative interviewers either in a conference room at the recruiting clinic (for participants recruited through Raleigh Neurology Associates) or at a hotel conference room near their treating clinic (for participants recruited through Global Market Research Group). The lead interviewer was a licensed clinical psychologist who co-facilitated all interviews, along with one other experienced interviewer who had a PhD in nursing. The interview participants were informed of the study objectives at the beginning of the interviews. All interviews were audio-recorded and transcribed for analysis.

### Analyses

Thematic analyses of the qualitative data were conducted to identify dominant trends in each interview and comparison across subsequent interviews to generate themes or patterns in the description of AD symptoms, impacts, and desired treatment outcomes. Specifically, interview transcripts were analyzed using ATLAS.ti and compared with the interviewers’ field notes, version 7.5 qualitative software (Berlin, Germany: Scientific Software Development; 2012). Each interview transcript was coded according to a qualitative coding frame, which was developed from the final interview guides and was refined iteratively to include additional codes as new concepts or themes were identified during review and analysis of the transcripts. To ensure consistency in coding, approximately 10% of the transcripts were double coded (i.e., two different interviewers coded these transcripts). Any discrepancies found between these codes were resolved by the two coders in discussion with the lead interviewer. The coded transcripts were reviewed and analyzed by the study team members.

## Results

### Participant characteristics

Patient demographic data were collected during screening for a total of 60 patients (Table 1). The majority of the patient sample was Caucasian (*n* = 46; 76.7%), with an average age of 72 years (range, 52–89 years), and the sample was evenly split between sexes (males, *n* = 27; 45.0%; females, *n* = 33; 55.0%). Most patients were retired (*n* = 38; 63.3%), married (*n* = 36; 60.0%), and referred for the study by primary care/general practitioners (*n* = 36; 60.0%). Almost three-quarters (*n* = 42; 70.0%) of the 60 patient participants in this study had a family history or friend with AD, with all but one group 1 participant (*n* = 11) reporting such a history. The majority of patients in the study were living with their spouse or partner (*n* = 28; 46.7%) or with their children (*n* = 11; 18.3%). All participants in groups 1, 2, and 3 were living independently (i.e., not in an assisted-living environment), and nearly half (*n* = 17; 47.2%) worked full- or part-time. Care partners reported that none of the patients in groups 4 and 5 were employed or living alone in their own home, and three participants (group 4, *n* = 1; group 5, *n* = 2) lived in an assisted-living facility.

**Table 1:** Patient characteristics

Patient characteristic	AD classification					Total patient sample (***N*** = 60)
	Group 1 (***n*** = 12)	Group 2 (***n*** = 12)	Group 3 (***n*** = 12)	Group 4 (***n*** = 12)	Group 5 (***n*** = 12)	
**Current age (years)**						
Mean (SD)	65.9 (10.3)	67.8 (8.8)	73.1 (10.1)	74.2 (7.6)	78.1 (9.3)	71.8 (10.0)
Range	56–89	53–82	52–87	60–89	57–88	52–89
**Sex,*n*(%)**						
Male	5 (41.7)	4 (33.3)	7 (58.3)	5 (41.7)	6 (50.0)	27 (45.0)
Female	7 (58.3)	8 (66.7)	5 (41.7)	7 (58.3)	6 (50.0)	33 (55.0)
**Race/ethnicity,*n*(%)**						
White/Caucasian	9 (75.0)	8 (66.7)	10 (83.3)	10 (83.3)	9 (75.0)	46 (76.7)
Black/African American	2 (16.7)	1 (8.3)	2 (16.7)	1 (8.3)	2 (16.7)	8 (13.3)
Asian	0 (0.0)	2 (16.7)	0 (0.0)	1 (8.3)	0 (0.0)	3 (5.0)
Hispanic or Latino	1 (8.3)	1 (8.3)	0 (0.0)	0 (0.0)	1 (8.3)	3 (5.0)
**Current employment status,*n*(%)**						
Employed full-time	5 (41.7)	4 (33.3)	3 (25.0)	0 (0.0)	0 (0.0)	12 (20.0)
Employed part-time	3 (25.0)	0 (0.0)	2 (16.7)	0 (0.0)	0 (0.0)	5 (8.3)
Retired	3 (25.0)	7 (58.3)	7 (58.3)	11 (91.7)	10 (83.3)	38 (63.3)
Disabled	0 (0.0)	0 (0.0)	0 (0.0)	0 (0.0)	2 (16.7)	2 (3.3)
Unemployed	1 (8.3)	1 (8.3)	0 (0.0)	1 (8.3)	0 (0.0)	3 (5.0)
**Highest level of education,*n*(%)**						
Less than high school	0 (0.0)	0 (0.0)	1 (8.3)	0 (0.0)	1 (8.3)	2 (3.3)
High school diploma or equivalent (GED)	0 (0.0)	1 (8.3)	2 (16.7)	5 (41.7)	5 (41.7)	13 (21.7)
Associate’s degree/technical school	2 (16.7)	0 (0.0)	2 (16.7)	0 (0.0)	0 (0.0)	4 (6.7)
Some college	5 (41.7)	4 (33.3)	1 (8.3)	3 (25.0)	3 (25.0)	16 (26.7)
College degree	4 (33.3)	7 (58.3)	4 (33.3)	4 (33.3)	2 (16.7)	21 (35.0)
Graduate or professional degree	1 (8.3)	0 (0.0)	2 (16.7)	0 (0.0)	1 (8.3)	4 (6.7)
**Current marital status,*n*(%)**						
Single	3 (25.0)	1 (8.3)	1 (8.3)	0 (0.0)	0 (0.0)	5 (8.3)
Married	8 (66.7)	8 (66.7)	6 (50.0)	8 (66.7)	6 (50.0)	36 (60.0)
Living with partner	0 (0.0)	0 (0.0)	1 (8.3)	0 (0.0)	0 (0.0)	1 (1.7)
Divorced or separated	0 (0.0)	1 (8.3)	2 (16.7)	0 (0.0)	1 (8.3)	4 (6.7)
Widowed	1 (8.3)	2 (16.7)	2 (16.7)	4 (33.3)	5 (41.7)	14 (23.3)
**Current living arrangement,*n*(%)**						
Alone, in own home or apartment	4 (33.3)	1 (8.3)	1 (8.3)	0 (0.0)	1 (8.3)^a^	7 (11.7)
With only spouse/partner	6 (50.0)^b^	6 (50.0)^c^	5 (41.7)	8 (66.7)	3 (25.0)	28 (46.7)
With only children	0 (0.0)	2 (16.7)^d^	2 (16.7)	3 (25.0)	4 (33.3)	11 (18.3)
With spouse/partner and child (ren)	2 (16.7)	2 (16.7)	3 (25.0)	0 (0.0)	2 (16.7)	9 (15.0)
With another relative	0 (0.0)	1 (8.3)^e^	1 (8.3)^f^	0 (0.0)	0 (0.0)	2 (3.3)
In an assisted-living facility	0 (0.0)	0 (0.0)	0 (0.0)	1 (8.3)	2 (16.7)	3 (5.0)
**Physician referral**						
Neurologist	1 (8.3)	2 (16.7)	2 (16.7)	5 (41.7)	5 (41.7)	15 (25.0)
Primary care/general practitioner	10 (83.3)	9 (75.0)	7 (58.3)	5 (41.7)	5 (41.7)	36 (60.0)
Other^g^	1 (8.3)	1 (8.3)	3 (25.0)	2 (16.7)	2 (16.7)	9 (15.0)
**Comorbidities,*n*(%)**						
Hypertension	2 (16.7)	2 (16.7)	5 (41.7)	2 (16.7)	3 (25.0)	14 (23.3)
Type 2 diabetes	1 (8.3)	3 (25.0)	1 (8.3)	2 (16.7)	3 (25.0)	10 (16.7)
High cholesterol	3 (25.0)	4 (33.3)	1 (8.3)	1 (8.3)	1 (8.3)	10 (16.7)
Depression	1 (8.3)	1 (8.3)	1 (8.3)	2 (16.7)	2 (16.7)	7 (11.7)
Other cardiovascular diseases^h^	1 (8.3)	1 (8.3)	1 (8.3)	1 (8.3)	3 (25.0)	7 (11.7)
Osteoporosis	2 (16.7)	2 (16.7)	0 (0.0)	1 (8.3)	2 (16.7)	7 (11.7)
Anxiety	2 (16.7)	1 (8.3)	0 (0.0)	2 (16.7)	1 (8.3)	6 (10.0)
Thyroid disease	0 (0.0)	2 (16.7)	0 (0.0)	1 (8.3)	1 (8.3)	4 (6.7)
Sleep apnea	0 (0.0)	1 (8.3)	0 (0.0)	2 (16.7)	0 (0.0)	3 (5.0)
Rheumatoid arthritis	1 (8.3)	0 (0.0)	1 (8.3)	1 (8.3)	0 (0.0)	3 (5.0)
Osteoarthritis	1 (8.3)	0 (0.0)	0 (0.0)	0 (0.0)	1 (8.3)	2 (3.3)
Gout	1 (8.3)	0 (0.0)	0 (0.0)	0 (0.0)	1 (8.3)	2 (3.3)
Glaucoma	1 (8.3)	0 (0.0)	0 (0.0)	0 (0.0)	0 (0.0)	1 (1.7)
COPD	0 (0.0)	1 (8.3)	0 (0.0)	0 (0.0)	0 (0.0)	1 (1.7)

*AD* Alzheimer’s disease, *COPD* chronic obstructive pulmonary disease, *GED* general educational development, *SD* standard deviation

Note: Demographic data for patients in groups 1, 2, and 3 were self-reported; data for patients in groups 4 and 5 were reported by care partners at screening

^a^This patient was currently living in an apartment for seniors but going to an assisted-living facility within the next few months

^b^One participant lived in a retirement village with his spouse

^c^One participant was living with her spouse and friends

^d^One participant reported that her son stays over at the home on occasion

^e^This participant was living with grandchildren

^f^This participant was living with his mother who had a stroke and providing care to her

^g^Other physician referrals were all from the Raleigh Neurology Associates Research Department except for one participant in group 4, who was referred via a psychiatrist

^h^Including heart attack, angina, coronary artery disease, congestive heart failure, peripheral vascular disease, cerebrovascular disease, and ischemic heart disease

The primary focus of interviews with 24 care partners (groups 4 and 5) was to understand how AD impacts the care partners, although some care partners described these impacts in terms of the needs of their loved one. The mean age of care partners was 60 years (range, 32–83 years), with the majority being female (*n* = 18; 75.0%) and Caucasian (*n* = 20; 83.3%) (Table 2). Approximately half of the care partners were caring for a parent (*n* = 13; 54.2%) or a spouse (*n* = 10; 41.7%). Care partners reported spending an average of 65 h (range, 8–168 h) in a typical week providing care. Most care partners were either employed full-time (*n* = 10; 41.7%) or retired (*n* = 9; 37.5%). Two were unemployed, and one was a student caring for a relative.

**Table 2:** Care partner characteristics

Demographic characteristic	AD classification		Overall sample (***n*** = 24)
	Group 4 (***n*** = 12)	Group 5 (***n*** = 12)	
**Current age (years)**			
Mean (SD)	64.1 (13.9)	56.0 (13.5)	60.0 (14.0)
Range	39–81	32–83	32–83
**Sex,*n*(%)**			
Male	3 (25.0)	3 (25.0)	6 (25.0)
Female	9 (75.0)	9 (75.0)	18 (75.0)
Relationship to patient, *n* (%)			
Child	4 (33.3)	9 (75.0)	13 (54.2)
Spouse	7 (58.3)	3 (25.0)	10 (41.7)
Cousin	1 (8.3)	0 (0.0)	1 (4.2)
Hours spent providing direct care to patient in a typical week			
Mean (SD)	65.7 (44.8)	65.3 (39.6)	65.7 (41.4)
Range	8–168	25–168	8–168
**Race/ethnicity,*n*(%)**			
White/Caucasian	10 (83.3)	10 (83.3)	20 (83.3)
Black/African American	1 (8.3)	2 (16.7)	3 (12.5)
Asian	1 (8.3)	0 (0.0)	1 (4.2)
**Current employment status,*n*(%)**			
Employed full-time	4 (33.3)	6 (50.0)	10 (41.7)
Employed part-time	0 (0.0)	2 (16.7)	2 (8.3)
Student	0 (0.0)	1 (8.3)	1 (4.2)
Retired	6 (50.0)	3 (25.0)	9 (37.5)
Unemployed (e.g., homemaker, not looking for work)	2 (16.7)	0 (0.0)	2 (8.3)
**Highest level of education,*n*(%)**			
High school diploma or equivalent (GED)	4 (33.3)	1 (8.3)	5 (20.8)
Associate’s degree/technical school	0 (0.0)	1 (8.3)	1 (4.2)
Some college	1 (8.3)	4 (33.3)	5 (20.8)
College degree	5 (41.7)	6 (50.0)	11 (45.8)
Graduate or professional degree	2 (16.7)	0 (0.0)	2 (8.3)

*AD* Alzheimer’s disease, *GED* general educational development, *SD* standard deviation

### Current AD symptoms

Patient self-reports and care partner reports indicated that all patients, including those in group 1, experienced at least one AD-related symptom at the time of the interviews.

#### Memory/forgetfulness

Participants in both early and late stages of AD experienced or were observed to have issues related to memory/forgetfulness (*n* = 60; 100.0%), including short-term memory issues, losing or misplacing things, and long-term memory issues (Table 3).

**Table 3:** Current AD symptoms reported in each group and overall: memory and forgetfulness

AD symptom, ***n*** (%)	AD classification					Overall sample (***N*** = 60)
	Group 1 (***n*** = 12)	Group 2 (***n*** = 12)	Group 3 (***n*** = 12)	Group 4 (***n*** = 12)	Group 5 (***n*** = 12)	
**Memory/forgetfulness**	**12 (100.0)**	**12 (100.0)**	**12 (100.0)**	**12 (100.0)**	**12 (100.0)**	**60 (100.0)**
Short-term memory issues	9 (75.0)	10 (83.3)	11 (91.7)	12 (100.0)	12 (100.0)	54 (90.0)
Losing or misplacing things	10 (83.3)	10 (83.3)	10 (83.3)	10 (83.3)	12 (100.0)	52 (86.7)
Relying more on lists, reminders, or other people	8 (66.7)	12 (100.0)	8 (66.7)	11 (91.7)	11 (91.7)	50 (83.3)
Forgetting dates or appointments	3 (25.0)	6 (50.0)	9 (75.0)	11 (91.7)	12 (100.0)	41 (68.3)
Forgetting why you walked into a room	8 (66.7)	7 (58.3)	7 (58.3)	7 (58.3)	11 (91.7)	40 (66.7)
Long-term memory issues	4 (33.3)	8 (66.7)	4 (33.3)	8 (66.7)	9 (75.0)	33 (55.0)
Forgetting to take medications	1 (8.3)	2 (16.7)	5 (41.7)	10 (83.3)	10 (83.3)	28 (46.7)
Forgetting to turn off running water or appliances	2 (16.7)	4 (33.3)	3 (25.0)	6 (50.0)	8 (66.7)	23 (38.3)
Forgetting to pay bills on time	2 (16.7)	1 (8.3)	2 (16.7)	7 (58.3)	10 (83.3)	22 (36.7)
Putting things in the wrong (inappropriate) place^a^	0 (0.0)	2 (16.7)	3 (25.0)	4 (33.3)	5 (41.7)	14 (23.3)

*AD* Alzheimer’s disease

Note: Information was collected from patients only from groups 1, 2, and 3; from care partners and patients (when able to self-report) in group 4; and from care partners only in group 5. Data shown are the number and percentage of individuals endorsing a symptom in each group and overall

^a^The item “Putting things in the wrong (inappropriate) place” was added after the first round of interviews was conducted, as this was an important item addressed by participants during the first round. The first round included 13 participants (group 1, *n* = 3; group 2, *n* = 2; group 3, *n* = 3; group 4, *n* = 3; group 5, *n* = 2)

Specifically, at least three-quarters of the patients in each group experienced general short-term memory issues (*n* = 54; 90.0% for overall sample) and losing or misplacing things (*n* = 52; 86.7%), with a similar frequency of reports in each group. Long-term memory issues were reported for more than half of the overall sample (*n* = 33, 55%). At least two-thirds of the patients in each of the five AD groups also relied more on lists, reminders, or other people (*n* = 50; 83.3%), and at least half of the participants in each group reported forgetting why they walked into a room (*n* = 40; 66.7%).

Although patients in the earlier AD groups reported some issues with memory/forgetfulness, the kind of issues reported and the frequency with which they were reported varied. Participants at risk for AD (groups 1 and 2) described short-term memory issues such as forgetting where they placed common objects, what they were supposed to buy (e.g., at the grocery store), and the need to use lists or other reminders. Participants in group 3 reported similar issues. However, more group 3 participants also more frequently reported forgetting dates or appointments, why they walked into a room, or to take medications. Short-term memory issues observed in individuals in later stages of the disease (groups 4 and 5) were largely related to the need for care partners to constantly repeat themselves to their care recipients or care recipients forgetting important tasks like turning off the water or stove.

The following selected quotations describe patients’ and care partners’ experiences with memory issues:

- ▪ “Sometimes I do [forget the names of people]. I meet people and I just try to remember their names … eventually I figure it out who they are.” (patient, group 1, in-depth interview [IDI] 6)
- ▪ “I just lose things … money … I can’t remember where I put things. I purposely tell myself, ‘Okay, I’m going to put this over here in a safe spot.’ And then when I need it, I don’t remember where I put it.” (patient 2, group 2, IDI 2)
- ▪ “I forget to turn off the lights … and lock the door and put out the garbage. It’s just general things.” (patient 1, group 3, IDI 1)
- ▪ “I don’t remember eating breakfast this morning. I don’t remember [what I had for lunch].” (patient 2, group 3, IDI 2)
- ▪ “We noticed … that she couldn’t remember how to get places that she had always gone really regularly. She had been coming to our house, got lost in the neighborhood, had to ask a neighbor how to get to the house.” (care partner 3, group 4, IDI 3)
- ▪ “Not being able to find normal things he [care recipient] should have. His watch, he has a very nice watch. Where did it go? ‘I don’t know. I had it on. Can’t find it.’ Those types of things …” (care partner, group 5, IDI 2)
- ▪ “She calls my brother me, and calls me my brother, and forgets who some people are …” (care partner, group 5, IDI 4)

#### Communication and language

Across all five AD groups, nearly all patients experienced symptoms related to communication and language (*n* = 55; 91.7%) (see Table S-1 in the Supplemental Appendix). A majority of patients in all groups reported or were observed to show such symptoms, with more than half in each of the five groups having difficulty finding the right words or names of things (*n* = 47; 78.3% for the overall sample) or losing their train of thought in conversation (*n* = 42; 70.0%). In addition, nearly half of patients (*n* = 26; 43%) were reported to have difficulty following what others were saying.

Group-level differences in symptoms related to communication and language were observed, beginning with group 3. At least half of the patients in groups 3, 4, and 5, compared with a quarter or less of patients in groups 1 and 2, were reported to have difficulty following what others were saying in conversations. Similarly, while at least one patient from each group was reported to not make sense to others when speaking, this experience was more commonly observed in groups 4 and 5. Patients in earlier stages of the disease noted that it sometimes took longer to recall people’s names and/or the names of objects, whereas participants in later stages forgot the names of common objects. Based on care partner reports, more patients in later stages of the disease (groups 4 and 5) exhibited difficulty following a conversation (e.g., not being able to attend to the conversation at hand, introducing an unrelated topic during a conversation). More than half of the group 4 and 5 care partners also reported that their care recipients would not make sense when speaking, sometimes using “gibberish.”

The following selected quotations describe patients’ and care partners’ experiences with communication and language issues:

- ▪ “I think it’s harder to come up with people’s names and street names and stuff like that.” (patient, group 1, IDI 4)
- ▪ “Oh yeah, losing track of what I’m talking about. I think it’s happening a lot more.” (patient, group 2, IDI 2)
- ▪ “I could be talking to you and I’m trying to pronounce ‘demonstrative’ and I might forget the word.” (patient, group 3, IDI 12)
- ▪ “Well, I’ll say … to tell him [care partner] get something like this out of the cabinet … And then I’ll say, ‘You know, it’s over there.’ [Cannot find the word for what is wanted] And he’ll say, ‘Well … I don’t know. Pancakes or something?’ I said, ‘No, you know. It’s in the bottle over there.’ I do that a lot.” (patient, group 4, IDI 6)
- ▪ “Sometimes he [care recipient] can talk normally. And then other times he’ll say instead of Ottawa, Ottawatawatawa. There’s really no train of thought anymore.” (care partner, group 5, IDI 1)

#### Other AD symptoms

Other AD symptoms reported fell into the categories of concentration and clear thinking, planning and organizing, orientation, changes in personality or behavior, and dependence (see Table S-2–Table S-7 in the Supplemental Appendix). Issues related to concentration and clear thinking for patients were reported by all care partners (100%) whose care recipients were in later stages of the disease (groups 4 and 5) and by nearly half of patients across groups 1, 2, and 3 (range, 41.0–50.0%). In addition, symptoms of AD related to planning and organizing and to orientation were reported by all of the care partners in group 5, almost all of the care partners in group 4 (*n* = 11; 91.7% for planning and organizing; *n* = 10; 83.3% for orientation) and by approximately half of the patients in group 3 (*n* = 6; 50.0% for planning and organizing; *n* = 7; 58.3% for planning and organizing). However, fewer than half of the patients in groups 1 and 2 reported symptoms in these two domains. Changes in patient behavior or personality were observed by almost all of the participants in groups 4 and 5 (*n* = 11; 91.7% for each group) and by approximately half of the participants in groups 1, 2, and 3 (range, 41.7–66.7%). All of the care partners in group 5 and almost all of the participants in group 4 (*n* = 11; 91.7%) reported that patients experienced AD symptoms related to dependence on others. Fewer patients in groups 1, 2, and 3 (range, 1–4 patients per group) reported issues with being dependent on others, and most of these participants noted that these issues were specifically related to driving.

### Frequency of symptoms by category

**Fig. 1:**
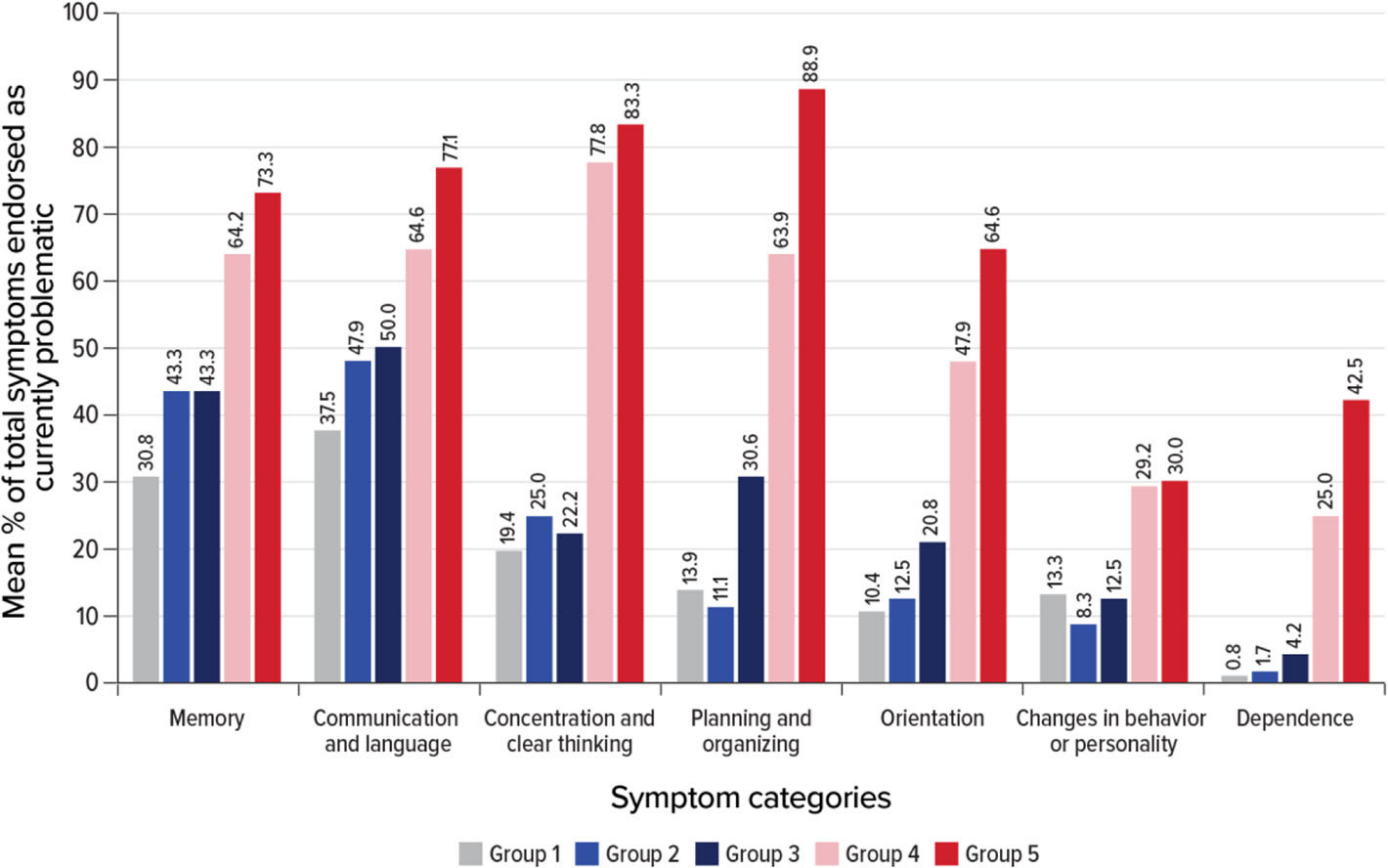
Mean percentage of items endorsed as currently problematic within a symptom category, by group. Note: Data shown are the mean number of symptoms reported by patients by group over the total number of symptoms in that category

To characterize the number of symptoms each patient experienced in a particular symptom category, the mean number of symptoms by the AD group was calculated and converted into a percentage of items endorsed. As shown in Fig. 1, current symptoms related to memory and to communication/language were endorsed even by participants who had not been diagnosed with AD and those in the early stage of AD (i.e., groups 1 and 2). However, the average frequency with which these issues were reported was generally seen to increase as patients moved into more advanced disease stages (groups 3, 4, and 5).

### Most bothersome or challenging issues

Patients and care partners were asked to describe the most bothersome or challenging aspect of AD-related symptoms. While some participants pointed to a particular symptom as being bothersome or challenging, others reported that concerns about the future bothered or challenged them most. Only two categories were identified by more than 40% of the sample as being most bothersome or challenging: memory/forgetfulness (short-term memory, forgetting friends and family, general memory issues, and losing or misplacing things) and “other” concerns such as uncertainty about the patient declining in the future and patients’ worry about being a burden on others for care (Table 4).

**Table 4:** Frequency of the most bothersome or challenging issues to patients and care partners

Most bothersome or challenging issue category, ***n*** (%)	AD classification					Total (***N*** = 60)
	Group 1 (***n*** = 12)	Group 2 (***n*** = 12)	Group 3 (***n*** = 12)	Group 4 (***n*** = 12)	Group 5 (***n*** = 12)	
**Memory/forgetfulness** (includes short-term memory; losing or misplacing things; forgetting medications; forgetting why you walked into a room; forgetting to turn off water or appliances; relying on lists, reminders, or others; long-term memory, other/general memory issues, forgetting friends/family)	7 (58.3)	5 (41.7)	5 (41.7)	7 (58.3)	1 (8.3)	25 (41.7)
**Other**^a^	3 (25.0)	1 (8.3)	5 (41.7)	9 (75.0)	7 (58.3)	25 (41.7)
**Changes in behavior or personality** (includes angry outbursts [getting mad], being impatient/irritable, being suspicious, anxiety, feeling scared, not wanting to do things you enjoyed before, feeling depressed or sad, feeling frustrated or flustered, getting upset, and other behavior or personality changes [*n* = 3])	1 (8.3)	1 (8.3)	3 (25.0)	7 (58.3)	2 (16.7)	14 (23.3)
**Dependence** (includes not being able to care for yourself, not being able to drive, and needing to move out of your home)	2 (16.7)	1 (8.3)	1 (8.3)	1 (8.3)	3 (25.0)	8 (13.3)
**Communication and language** (includes difficulty following conversations, losing train of thought, difficulty finding words and names, not making sense when speaking)	2 (16.7)	1 (8.3)	1 (8.3)	2 (16.7)	1 (8.3)	7 (11.7)
**Concentration and clear thinking** (includes difficulty focusing/paying attention, difficulty managing money, difficulty making decisions)	2 (16.7)	3 (25.0)	1 (8.3)	0 (0.0)	0 (0.0)	6 (10.0)
**Awareness of day/time/people** (includes knowing where you are and knowing the direction you are going)	1 (8.3)	2 (16.7)	1 (8.3)	0 (0.0)	0 (0.0)	4 (6.7)
**Planning** (includes difficulty understanding instructions)	0 (0.0)	0 (0.0)	0 (0.0)	0 (0.0)	1 (8.3)	1 (1.7)

*AD* Alzheimer’s disease

Note: Information was collected from patients only from groups 1, 2, and 3; from care partners and patients (when able to self-report) in group 4; and from care partners only in group 5. Data shown are the number and percentage of individuals endorsing a symptom in each group and overall

^a^The “other” category included issues such as what the future holds/uncertainty/noticing a decline (*n* = 7), patients feeling as though they are or could become a burden (*n* = 6), care partners’ need to repeating themselves (*n* = 4), care partners’ frustration/needing patience (*n* = 2), care partners’ difficulty keeping on top of patient and family needs and schedules (*n* = 2), the patient’s inability “to do anything” (*n* = 1), safety issues (*n* = 1), and the patient sleeping a lot (*n* = 1)

### Impacts

#### Impacts to patients (groups 1, 2, and 3)

When asked to describe the impacts of their AD-related symptoms, all patients in groups 1, 2, and 3 reported experiencing one or more impacts (Table 5). Emotional impact and social impacts were the most commonly reported across all three groups, with both impacts reported more frequently in group 3 than in groups 1 and 2.

**Table 5:** Frequency of impacts reported by patients in groups 1, 2, and 3

Impact, ***n*** (%)	AD classification			Total (***n*** = 36)
	Group 1 (***n*** = 12)	Group 2 (***n*** = 12)	Group 3 (***n*** = 12)	
**Mood or emotions have changed** (e.g., is more frustrated, stressed, anxious, worried, impatient/irritable, angry, scared/frightened, overwhelmed, or sad/depressed)	8 (66.7)	6 (50.0)	9 (75.0)	23 (63.9)
**Social activities or outgoingness have decreased** (e.g., limits where he or she will go, prefers to stay at home, is less outgoing)	4 (33.3)	7 (58.3)	6 (50.0)	17 (47.2)
**Daily activities have become more difficult to complete** (e.g., has difficulty with cooking, performing household chores, running errands)	3 (25.0)	3 (25.0)	6 (50.0)	12 (33.3)
**Working has become impossible or more challenging** (e.g., stopped working or works less, has increased challenges or stress with work tasks and may have changed tasks to things that were easier to do/not as challenging for memory, planning to retire in the near future)	4 (33.3)	3 (25.0)	3 (25.0)	10 (27.8)
**Leisure activities have decreased or ceased** (e.g., stopped or limits hobbies, travel, volunteering)	5 (41.7)	1 (8.3)	4 (33.3)	10 (27.8)
**Reliance on care partner has increased**	0 (0.0)	3 (25.0)	6 (50.0)	9 (25.0)
**Other**^**a**^	2 (16.7)	5 (41.7)	1 (8.3)	8 (22.2)
**Driving has become impossible or more challenging** (e.g., gets lost while driving, has trouble with night driving, does not drive far distances, needs to drive less or stop driving, uses more public transportation instead of driving)	1 (8.3)	2 (16.7)	3 (25.0)	6 (16.7)
**Future plans and arrangements have been made** (e.g., has performed estate planning, retirement, living arrangements, financial plans)	3 (25.0)	2 (16.7)	1 (8.3)	6 (16.7)
**Sleep is disturbed**	3 (25.0)	1 (8.3)	2 (16.7)	6 (16.7)
**Assistance with finances or paying bills is needed**	0 (0.0)	2 (16.7)	2 (16.7)	4 (11.1)
**Assistance with personal hygiene is needed** (e.g., requires help with showering and bathing)	0 (0.0)	0 (0.0)	2 (16.7)	2 (5.6)
**Living full-time with family other than spouse/partner has become necessary**	0 (0.0)	0 (0.0)	1 (8.3)	1 (2.8)

Note: Data shown are the number and percentage of individuals endorsing an impact in each group and overall

^a^The “other” category included impacts such as everything happening at a slower pace (*n* = 2 [group 1], *n* = 1 [group 3]), feeling like there is nothing he or she can do to make things better (*n* = 1 [group 1]), not feeling comfortable going places at night (*n* = 1 [group 1]), needing to explain what he or she wants to say when unable to find the right word (*n* = 1 [group 1]), checking to make sure appliances and lights are not left on (*n* = 1 [group 1]), running late due to misplacing things (*n* = 1 [group 1]), handwriting has declined (*n* = 1 [group 2]), feeling that life is different in general (*n* = 1 [group 2]), being unable to enjoy going and doing things due to forgetting about other responsibilities (*n* = 1 [group 2]), needing to stick to familiar tasks and places (*n* = 1 [group 2]), thinking and talking things through a little bit more (*n* = 1 [group 2]), needing to be more organized and aware (*n* = 1 [group 2]), and needing to be more cautious (*n* = 1 [group 2])

More than half of the sample in groups 1, 2, and 3 reported an impact on mood and emotions (*n* = 23; 63.9%). Nearly half of the overall patient sample (*n* = 17; 47.2%) and at least half of the patients in group 2 (*n* = 7; 58.3%) and group 3 (*n* = 6; 50.0%) reported an impact on their social life/activities. Furthermore, the number of patients reporting AD-related impacts on daily activities, increased reliance on care partners, and increased need for assistance with personal hygiene was greater in group 3 than in groups 1 and 2. The following selected quotations describe the impact of AD-related symptoms for patients in these groups:

- ▪ “[Difficulty with quick recall] is very frustrating and it has changed my mood. I’m not happy about it … Physically I’m fine. But I mean, you know, if your mind is not good, then there’s no point. … I think the inward frustration leads to depression, which is why I’m taking Lexapro right now. Because I have anxiety over this thing. So I’m just hoping it’s just natural aging or … I know it’s not going to get better because plaque is plaque or whatever. But I would hope it would.” (patient, group 1, IDI 3)
- ▪ “It [current symptoms] just makes me very anxious. It makes me think of all the things that I used to be able to do well and I don’t do so well now. So that gets me really down. I just don’t want to be a burden.” (patient, group 2, IDI 2)
- ▪ “Well, it [difficulty concentrating/focusing] frustrates you. It overwhelms you. And you’re like, ‘Am I crazy?’ And I say, ‘Why is it that I, nobody else has a problem with this, but I have a problem with this. Why can’t I get this together?’” (patient, group 3, IDI 5)
- ▪ “I find myself being a little bit more of a homebody than before. I used to go out all the time and do things. So I just find myself staying at home more. It sounds kind of crazy, but … sometimes I don’t feel comfortable going places at night like I used to and stuff.” (patient, group 1, IDI 12)
- ▪ “I guess maybe [I am] going out less with other couples because I don’t want them really to know. So I guess I kind of stay back and away. Maybe they would start talking about something we did and I wouldn’t remember … I really don’t want to go.” (patient, group 2, IDI 2)
- ▪ “Well, I do get sad about my situation sometimes. I don’t think it causes me to withdraw that much, but I’m not as outgoing as I used to be. I used to do a lot of caregiving for others, and I’m not doing that right now. I don’t feel responsible enough … I need to take care … keep up with what I’m doing.” (patient, group 3, IDI 8)

#### Impacts to care partners (groups 4 and 5)

Although care partners and patients (when they were capable of reporting on their own experiences) in group 4 both reported on patients’ symptoms, care partners in groups 4 and 5 were asked to report on the ways in which AD had impacted their own lives versus the impact of AD on their care recipients (Table 6). All 24 care partners (100.0%) reported being impacted in some way by caring for an individual with AD, with care partners unanimously reporting impacts on their own daily responsibilities (e.g., the need to supervise or drive the patient, changing roles, impacts on daily chores such as cooking). Other commonly reported impacts included mood and emotional impacts on the care partner (*n* = 19; 79.2%); being less social (*n* = 19; 79.2%); decreasing or ceasing leisure activities (e.g., hobbies, travel, going out to eat, volunteering) (*n* = 18; 75.0%); managing the patient’s money and/or taking on increasing financial responsibility (*n* = 18; 75.0%); performing or helping with the patient’s self-care, cooking, or taking medication (*n* = 16; 66.7%); experiencing physical health issues (*n* = 16; 66.7%); stopping the patient from driving (*n* = 14; 58.3%); reminding the patient about appointments, eating, taking medication, and showering (*n* = 13; 54.2%); needing to repeat things to the patient more frequently (*n* = 13; 54.2%); being concerned about the patient’s welfare (*n* = 13; 54.2%); and experiencing work/school disruptions (*n* = 12; 50.0%).

**Table 6:** Frequency of care partner impacts

Impact, ***n*** (%)	AD classification		Total (***n*** = 24)
	Group 4 (***n*** = 12)	Group 5 (***n*** = 12)	
**Daily responsibilities are impacted** (e.g., needs to supervise and drive patient more, roles have changed, daily chores [e.g., cooking] have been impacted)	12 (100.0)	12 (100.0)	24 (100.0)
Constant supervision of patient is required	10 (83.3)	7 (58.3)	17 (70.8)
**Mood or emotions have changed**	11 (91.7)	8 (66.7)	19 (79.2)
Feeling bad or guilty more frequently	5 (41.7)	2 (16.7)	7 (29.2)
**Social activities have decreased** (e.g., time with friends for lunch/dinner/something fun, parties/celebrations, and family events has decreased)	10 (83.3)	9 (75.0)	19 (79.2)
**Leisure activities have decreased or ceased** (e.g., hobbies, travel, going out to eat, or volunteering has decreased or ceased)	8 (66.7)	10 (83.3)	18 (75.0)
**Responsibilities for patient’s bills or money management have increased/financial burden has increased**	9 (75.0)	9 (75.0)	18 (75.0)
**Other**^**a**^	10 (83.3)	8 (66.7)	18 (75.0)
**Assistance with patient’s self-care, cooking, and medications has increased** (e.g., showering, making sure patient takes medication, toileting, cooking)	7 (58.3)	9 (75.0)	16 (66.7)
Assistance with toileting has increased (e.g., taking patient to bathroom, reminding him or her to use the bathroom, cleans up accidents related to incontinence)	1 (8.3)	4 (33.3)	5 (20.8)
**Physical health has suffered** (e.g., sleep, diet, exercise, weight gain)	7 (58.3)	9 (75.0)	16 (66.7)
**Prevention of patient driving has become necessary**	6 (50.0)	8 (66.7)	14 (58.3)
**Reminders to the patient are more frequent** (e.g., reminding more frequently to eat, take medications, and shower and that he/she has appointments)	9 (75.0)	4 (33.3)	13 (54.2)
**Repeating to the patient has become more frequent**	8 (66.7)	5 (41.7)	13 (54.2)
**Patient welfare has become an increasing concern** (safety concerns [e.g., starting a fire, wandering, house flooding] or fears or the patient being taken advantage of have increased)	9 (75.0)	4 (33.3)	13 (54.2)
**Working or schooling has become impossible or more challenging** (e.g., stopped working or decreased hours due to needing to supervise patient, needs to take off more from work/use vacation time/sick days to care for patient, needs to coordinate patient’s care when at work, needs to shift work hours to provide care for patient/needs a flexible job, needs to work or study at home, having trouble finding time to continue schooling)	7 (58.3)	5 (41.7)	12 (50.0)
**Planning and making decisions on behalf of the patient has become necessary**	8 (66.7)	3 (25.0)	11 (45.8)
**Moving in/living with the patient was required in order to provide more assistance**	3 (25.0)	3 (25.0)	6 (25.0)
**Use of locks, alarms, location-tracking apps, and/or cameras to keep patient safe/prevent disasters (e.g., fires and floods) has become necessary**	2 (16.7)	3 (25.0)	5 (20.8)
**In-home professional help is employed**	1 (8.3)	3 (25.0)	4 (16.7)
**Future plans and arrangements have been made** (e.g., estate planning, retirement, living arrangements, financial plans)	2 (16.7)	2 (16.7)	4 (16.7)

Note: Both patients and care partners in group 4 were asked about impacts; because patients were not able to reliably report on impacts, impacts to only care partners in group 4 are shown. Impacts shown are those reported by care partners in groups 4 and 5 who were asked to describe the impact of AD on their own lives and not the impact of AD on their care recipients. Data shown are the number and percentage of individuals endorsing an impact in each group and overall

^a^The “other” category included impacts such as having more general responsibility/picking up the slack (*n* = 3 [group 4]); concerns about getting AD as well (*n* = 2: group 4, *n* = 1; group 5, *n* = 1); needing to walk the patient through instructions (*n* = 2 [group 4]; *n* = 1 [group 5]); being harder for the patient to attend the care partner’s child’s activities (*n* = 1 [group 4]); being busy/having no time due to needing to go to doctor’s appointments (*n* = 1 [group 4]); trying to keep the patient calm (*n* = 1 [group 4]); developing routine at home/ways to keep the patient more organized (*n* = 1 [group 4]); monitoring changes per the Alzheimer’s Association list and reporting to doctors (*n* = 1 [group 4]); needing to be unselfish (*n* = 1 [group 4]); avoiding communication (*n* = 1 [group 4]); not being able to get the patient to agree to certain things (*n* = 1 [group 4]); feeling tired (*n* = 1 [group 4]); leaving the house messy (*n* = 1 [group 4]); having to answer questions from the patient that he or she previously would have known the answer to (*n* = 1 [group 4]); feeling as though they are a child again who needs to report to their parent, who is the care recipient (*n* = 1 [group 4]); feeling resentful of other sibling who does not help with caregiving (*n* = 1 [group 4]); needing help from brother, children, and friends for caretaking (*n* = 1 [group 4]); doing anything he or she can to make the patient happy and get the most out of life (*n* = 1 [group 4]); having a more scheduled lifestyle (*n* = 1 [group 4]); losing weight/becoming healthier (*n* = 1 [group 4]); unable to visit romantic partner who lives in another state (*n* = 1 [group 4]); finding it harder to interact with the patient due to him or her misinterpreting things (*n* = 1 [group 5]); finding it harder to do activities together as a family/children need to be more independent (*n* = 1 [group 5]); and feeling that things are unpredictable (*n* = 1 [group 5])

Most impacts were reported with similar frequency among care partners in groups 4 and 5, with a few notable exceptions. Compared with care partners in group 5, more care partners in group 4 reported impacts in the areas of mood or emotions, reminding or repeating information to their care recipient, concern for the care recipient’s welfare, and planning and decision-making. Compared with care partners to group 4 patients, fewer care partners in group 5 reminded patients to do things such as eat, take their medications, shower, and go to appointments; instead, it was noted that more care partners in group 5 (*n* = 9; 75.0%) than in group 4 (*n* = 7; 58.3%) actually helped patients perform these activities.

### Treatment-related outcomes

All participants were asked what an ideal treatment for AD would do for them (groups 1 through 4) or for their care recipient (groups 4 and 5) and/or what issues would be the most important for an AD treatment to address (Table 7). The most important outcomes for AD treatment fell into three primary categories: better memory functioning, disease modification, maintaining (or increasing) independence. Approximately 67% of the participants in this study (*n* = 40) wanted to see an improvement in or restoration of memory with an AD treatment. Additionally, over half of the participants in each of the five groups wanted a treatment to stop AD progression (*n* = 35; 58.3%).

**Table 7:** Treatment-related outcomes most important to patients with or at risk for AD and care partners

Ideal treatment outcome, ***n*** (%)	AD classification					Total (***N*** = 60)
	Group 1 (***n*** = 12)	Group 2 (***n*** = 12)	Group 3 (***n*** = 12)	Group 4 (***n*** = 12)	Group 5 (***n*** = 12)	
Improve/restore memory	9 (75.0)	7 (58.3)	5 (41.7)	9 (75.0)	10 (83.3)	40 (66.7)
Stop AD progression	8 (66.7)	6 (50.0)	7 (58.3)	7 (58.3)	7 (58.3)	35 (58.3)
Slow AD progression	4 (33.3)	6 (50.0)	3 (25.0)	5 (41.7)	2 (16.7)	20 (33.3)
Improve ability to function, perform ADLs	4 (33.3)	0 (0.0)	1 (8.3)	5 (41.7)	5 (41.7)	15 (25.0)
Improve short-term memory	3 (25.0)	3 (25.0)	3 (25.0)	3 (25.0)	2 (16.7)	14 (23.3)
Remember family	5 (41.7)	0 (0.0)	2 (16.7)	3 (25.0)	2 (16.7)	12 (20.0)
Cure AD	1 (8.3)	3 (25.0)	1 (8.3)	3 (25.0)	3 (25.0)	11 (18.3)
Help remain independent, not be a burden	1 (8.3)	0 (0.0)	0 (0.0)	2 (16.7)	3 (25.0)	6 (10.0)
Remove plaque/tangles/stop growth	2 (16.7)	0 (0.0)	1 (8.3)	0 (0.0)	3 (25.0)	6 (10.0)
Be sharper, more focused	0 (0.0)	3 (25.0)	0 (0.0)	1 (8.3)	1 (8.3)	5 (8.3)
Be aware of self and surroundings	0 (0.0)	0 (0.0)	0 (0.0)	1 (8.3)	3 (25.0)	4 (6.7)
Improve long-term memory	1 (8.3)	0 (0.0)	0 (0.0)	2 (16.7)	0 (0.0)	3 (5.0)
Stop hallucinations	0 (0.0)	0 (0.0)	0 (0.0)	1 (8.3)	0 (0.0)	1 (1.7)

*AD* Alzheimer’s disease, *ADLs* activities of daily living

Note: Participants were allowed to report multiple treatment outcomes; thus, the sums exceed 100%. Data shown are the number and percentage of individuals endorsing an outcome in each group and overall. Information was collected from patients only from groups 1, 2, and 3; from care partners and patients (when able to self-report) in group 4; and from care partners only in group 5

Improving or restoring memory was an important treatment outcome across all groups, as memory-related symptoms were prevalent from early to late stages of the disease. Participants noted that with improved or restored memory, other AD-related issues would subsequently improve; those in groups 1, 2, and 3 described how improved memory would allow them to continue to function as usual in daily life and to remember things without difficulty as they had prior to the onset of current concerns. For those in groups 4 and 5, improvement in memory meant being able to perform a broad spectrum of ADLs, such as independently cooking, bathing, or driving, as well as being able to know where they were and engage in the environment around them. The following selected quotations describe many participants’ views on the importance of slowing disease progression:

- ▪ “Definitely [meaningful to stop progression] because I still have a good life. I’m still able to enjoy my grandbabies; I’m still able to go places; I’m still … independent, bathe myself, do my hair if I want to … I’m still functioning very highly. But just to think that in 4 years [is concerning].” (patient, group 1)
- ▪ “If they could stop it right now, then I could continue to function like I am. If my processing time continues to slow down, then it makes it harder and harder to be in a conversation or to interact with people.” (patient, group 1)
- ▪ “The medication would slow down my forgetfulness … if it slowed it down I’d have more years to take care of myself and not have to worry about other people taking care of me.” (patient, group 2)
- ▪ “Just, if it can’t cure it, just kind of stop the progression of it. Just like leave if it where it is. If the worse thing I have is momentary lapses every few days, 3 days, and I’m still on the path I am now, I’m happy.” (patient, group 3)
- ▪ “Ideally it would just stop the memory loss. I could definitely live and survive the way I am now. But you don’t know what’s going to happen in the future, how it progresses.” (patient, group 3)
- ▪ “If you could just find a single medication that could stop her [care recipient] where she’s at right now, that would be even in itself a huge breakthrough. Because maybe we could figure out how to work out the issues that she does have.” (care partner, group 4)
- ▪ “That’s going to be valuable [to stop progression]. Like as of now, it’s fine … manageable. If they can stop now, I’m happy with that …” (care partner, group 4)
- ▪ “Because we’re doing really good right now. If it would stop today, I would probably try to figure out how to go on trips … whether he [care recipient] remembered people or not, that wouldn’t be the biggest deal about the medicine. If we were able to converse and understand each other and we could take trips and he could enjoy doing that.” (care partner, group 5)
- ▪ “[Stopping the progression] … means I don’t have to think about what happens in the future if he [care recipient] has to go to one of those homes who will take care of him.” (care partner, group 5)

## Discussion

Results from the current study suggest that issues with various aspects of cognitive functioning—particularly in memory and communication/language—are present even in the earliest stages, including individuals who have not received a formal diagnosis (groups 1 and 2) and those with mild AD (group 3). Per inclusion criteria, all group 1 participants should not have been showing signs of clinical impairment, which is consistent with our findings. However, all 12 group 1 patients reported some current “problem” with cognitive functioning. Further, all patients in groups 1, 2, and 3 experienced impacts related to AD symptoms, most commonly affecting their emotions and mood or their social lives, and the frequency of these impacts tended to increase with increasing AD severity. Care partners to patients in groups 4 and 5 also experienced impacts related to their care recipients’ AD symptoms—particularly impacts to the care partners’ daily responsibilities and their emotions and mood. Taken together, these findings illustrate that AD affects patients and their partners across the continuum disease stages, including in preclinical stages.

The data from group 3 participants (those with mild AD) were much more variable than the patterns seen in other groups. These findings suggest that a sample of individuals with mild AD is much more heterogenous than some of the other AD groups in the study. This broad heterogeneity may suggest differences in how clinicians view the diagnosis of mild AD and may indicate a need to assess differences in patients’ priorities within this group and not just between this group and other groups.

Across the AD continuum, improved memory, disease modification, and remaining independent (including in the ability to perform daily activities) were the AD treatment outcomes considered to be most important to individuals with or at risk for AD and to their care partners. Traditional neuropsychological assessments measure various aspects of cognitive functioning often impaired by AD, but it is unclear whether they can measure the often-subtle changes that are important to patients and care partners, particularly as their experiences and priorities evolve across the AD severity spectrum. Specifically, concepts such as decreased socialization due to concerns about forgetting names or losing track when speaking to others; mood-related symptoms such as depression or anxiety attributable to changes in memory; and increased dependence on others for even basic chores may be infrequently collected in neuropsychological assessments, especially in preclinical stages of AD [5]. Further, these concepts may represent previously underrecognized unmet needs for individuals with AD and their care partners and should be reflected in support strategies targeted toward the AD community. Data from the current study provide some evidence that changes seen in certain domains of traditional neuropsychological assessments (e.g., short-term memory) could be meaningful to individuals with or at risk for AD and their care partners. Future research should explore how the concepts of importance identified in this study are reflected in current measures of AD disease severity, functioning, and treatment outcomes to inform outcome assessment strategies and clinical trial design.

Worldwide efforts are underway to slow, delay, or stop AD progression. Measuring this requires some type of time-to-event analysis or assessment of change in disease course [3]. Results from the current study provide some evidence that if researchers were able to demonstrate an impact on disease course, it would be meaningful to both individuals with or at risk for AD and their care partners. Furthermore, delay in progression is likely an important outcome to payers and regulators, as a delay in institutional care could represent a substantial cost savings [13]. Given that study participants as early as in group 1 reported current concerns and related impacts, researchers face the challenge of better understanding the meaningfulness of traditional clinical measures, especially in individuals who may not yet show signs of MCI.

### Limitations

Some limitations of this study should be considered. First, while 60 in-depth interviews would be considered more than an adequate sample size in many qualitative studies, this sample was relatively small when split across five AD groups (12 per group). Thus, between-group comparisons and subgroup analyses of the data were limited. Additionally, as a function of the cognitive impairments associated with AD itself, patient self-report was limited to those in earlier or preclinical stages (groups 1–3). More than half of the patients and care partners were Caucasian, and almost all of the care partners were female. Thus, given the relative small sample size for each AD group and the limited representativeness of the participants in terms of demographic characteristics, it is not known how generalizable these findings are to the broader AD patient and care partner populations. This study was intended as a first step in understanding what matters most to individuals affected by AD, and additional research—especially in patients of different ethnicities and representing other subpopulations—is warranted to obtain knowledge on the potential differences among groups in terms of AD-related impacts and desired treatment outcomes. Further, there is potential for selection bias for patient participants in group 1, who, despite exhibiting no clinical impairment, had undergone testing for AD pathology, perhaps resulting from a family history of AD or some manifestation of the disease already present that had prompted them to get the test. This group may not be representative of all patients with positive amyloid positron emission tomography; moreover, comparative data from individuals who were sociodemographically matched to group 1 participants but exhibited no clinical AD pathology were not available. Thus, some of the symptoms reported by group 1 participants may be related to aging rather than AD pathology. Additionally, although the study was rigorously designed and all study participants were recruited for this study through clinicians using strict inclusion/exclusion criteria, interview data suggest that diagnostic practices may vary in the general population. Finally, the results are subject to volunteer and potential selection bias and may not be representative of a broader sample. The data that emerged from this qualitative study, albeit not confirmatory, established trends in the concepts of importance to patients and care partners affected by AD; future research is planned to explore the importance of these concepts in further detail and in additional and expanded populations.

## Conclusions

The current study aimed to examine what is important to individuals and care partners across the AD disease continuum. Issues with various aspects of cognitive functioning—particularly in memory and communication—are present even in individuals with preclinical AD. Improved memory and disease modification were treatment outcomes considered most important to participants across all five AD stages. Results from this study informed the development of a quantitative study, currently in progress, to elicit the relative importance of these concepts of interest to people at risk for and living with AD and their care partners. Collectively, the results of this research lay the foundation for the development of recommendations for clinical outcomes assessments in AD research studies.

## Supplementary information

**Additional file 1.** Supplemental Appendix

## Data Availability

The datasets generated and/or analyzed during the current study are not publicly available.

## Abbreviations

AD: Alzheimer’s disease
AD PACE: Alzheimer’s Disease Patient and Caregiver Engagement Initiative
ADAS-Cog: Alzheimer’s Disease Assessment Scale—Cognitive
ADLs: Activities of daily living
COPD: Chronic obstructive pulmonary disease
CSF: Cerebrospinal fluid
FDA: US Food and Drug Administration
GED: General educational development
MCI: Mild cognitive impairment
MMSE: Mini-Mental State Examination
SD: Standard deviation
US: United States

## References

[1] Alzheimer’s Association (2019). Medical tests.

[2] American Psychiatric Association (2013). Diagnostic and statistical manual of mental disorders.

[3] Food and Drug Administration (FDA). Early Alzheimer’s disease: developing drugs for treatment guidance for industry. Draft guidance. 2018. https://www.fda.gov/downloads/Drugs/GuidanceComplianceRegulatoryInformation/Guidances/UCM596728.pdf. Accessed 11 Feb 2019.

[4] Food and Drug Administration (FDA). Patient-focused drug development. Draft guidance. April 21, 2020. https://www.fda.gov/drugs/development-approval-process-drugs/cder-patient-focused-drug-development. Accessed 17 Jun 2020.

[5] Hartry A, Aldhouse NVJ, Al-Zubeidi T, Sanon M, Stefanacci RG, Knight SL (2018). The conceptual relevance of assessment measures in patients with mild/mild-moderate Alzheimer’s disease. Alzheimers Dement (Amst).

[6] Kueper JK, Speechley M, Montero-Odasso M (2018). The Alzheimer’s Disease Assessment Scale-Cognitive Subscale (ADAS-Cog): modifications and responsiveness in pre-dementia populations. A narrative review. J Alzheimers Dis.

[7] Mohs RC, Knopman D, Petersen RC, Ferris SH, Ernesto C, Grundman M (1997). Development of cognitive instruments for use in clinical trials of antidementia drugs: additions to the Alzheimer’s Disease Assessment Scale that broaden its scope. The Alzheimer’s Disease Cooperative Study. Alzheimer Dis Assoc Disord.

[8] Rosen WG, Mohs RC, Davis KL (1984). A new rating scale for Alzheimer’s disease. Am J Psychiatry.

[9] Saunders S, Muniz-Terrera G, Watson J, Clarke CL, Luz S, Evans AR (2018). Participant outcomes and preferences in Alzheimer’s disease clinical trials: the electronic Person-Specific Outcome Measure (ePSOM) development program. Alzheimers Dement (N Y).

[10] Sim J, Saunders B, Waterfield J, Kingstone T (2018). Can sample size in qualitative research be determined a priori?. Int J Social Research Methodol.

[11] Vasileiou K, Barnett J, Thorpe S, Young T (2018). Characterising and justifying sample size sufficiency in interview-based studies: systematic analysis of qualitative health research over a 15-year period. BMC Med Res Methodol.

[12] Watson J, Saunders S, Muniz Terrera G, Ritchie C, Evans A, Luz S (2019). What matters to people with memory problems, healthy volunteers and health and social care professionals in the context of developing treatment to prevent Alzheimer’s dementia? A qualitative study. Health Expect.

[13] Wübker A, Zwakhalen SM, Challis D, Suhonen R, Karlsson S, Zabalegui A (2015). Costs of care for people with dementia just before and after nursing home placement: primary data from eight European countries. Eur J Health Econ.

