# Supplementary material for: Assessing what matters most to patients with or at risk for Alzheimer’s and care partners: a qualitative study evaluating symptoms, impacts, and outcomes
Source: Alzheimers Res Ther. 2020 Jul 30;12:90. doi: 10.1186/s13195-020-00659-6 (PMC7393916; doi:10.1186/s13195-020-00659-6)
Supplement: Supplementary file 1 — Additional file 1. Supplemental Appendix [file 13195_2020_659_MOESM1_ESM.docx]

# Supplemental Appendix

1. Current AD Symptoms Reported In Each Group and Overall: Communication and Language

| AD Symptom, n (%) | AD Classification | | | | | Overall Sample (N = 60) |
| --- | --- | --- | --- | --- | --- | --- |
|  | Group 1 (n = 12) | Group 2 (n = 12) | Group 3 (n = 12) | Group 4 (n = 12) | Group 5 (n = 12) |  |
| Communication and language | 10 (83.3) | 12 (100.0) | 11 (91.7) | 10 (83.3) | 12 (100.0) | 55 (91.7) |
| Difficulty finding the right words or names of things | 7 (58.3) | 10 (83.3) | 9 (75.0) | 9 (75.0) | 12 (100.0) | 47 (78.3) |
| Losing train of thought in conversations, losing track of what you are talking about | 7 (58.3) | 9 (75.0) | 7 (58.3) | 9 (75.0) | 10 (83.3) | 42 (70.0) |
| Difficulty following what other people are saying in conversations | 3 (25.0) | 2 (16.7) | 6 (50.0) | 7 (58.3) | 8 (66.7) | 26 (43.3) |
| Not making sense to others when speaking | 1 (8.3) | 2 (16.7) | 2 (16.7) | 6 (50.0) | 7 (58.3) | 18 (30.0) |

AD = Alzheimer’s disease.

Note: Information was collected from patients only from Groups 1, 2, and 3; from care partners and patients (when able to self-report) in Group 4; and from care partners only in Group 5. Data shown are the number and percentage of individuals endorsing a symptom in each group and overall.

1. Current AD Symptoms Reported In Each Group and Overall: Changes in Behavior or Personality

| AD Symptom, n (%) | AD Classification | | | | | Overall Sample (N = 60) |
| --- | --- | --- | --- | --- | --- | --- |
|  | Group 1 (n = 12) | Group 2 (n = 12) | Group 3 (n = 12) | Group 4 (n = 12) | Group 5 (n = 12) |  |
| Changes in behavior or personality | 8 (66.7) | 5 (41.7) | 8 (66.7) | 11 (91.7) | 11 (91.7) | 43 (71.7) |
| Being impatient/irritable | 5 (41.7) | 3 (25.0) | 6 (50.0) | 9 (75.0) | 7 (58.3) | 30 (50.0) |
| Angry outbursts | 4 (33.3) | 2 (16.7) | 3 (25.0) | 8 (66.7) | 6 (50.0) | 23 (38.3) |
| Feeling scared | 3 (25.0) | 2 (16.7) | 2 (16.7) | 6 (50.0) | 9 (75.0) | 22 (36.7) |
| Not wanting to do things you enjoyed before | 3 (25.0) | 2 (16.7) | 1 (8.3) | 8 (66.7) | 7 (58.3) | 21 (35.0) |
| Being suspicious or not trusting family, friends, or care partner | 1 (8.3) | 1 (8.3) | 3 (25.0) | 4 (33.3) | 7 (58.3) | 16 (26.7) |

AD = Alzheimer’s disease.

Note: Information was collected from patients only from Groups 1, 2, and 3; from care partners and patients (when able to self-report) in Group 4; and from care partners only in Group 5. Data shown are the number and percentage of individuals endorsing a symptom in each group and overall.

1. Additional Spontaneously Reported Current AD Symptoms Reported In Each Group and Overall

| AD Symptom, n (%) | AD Classification | | | | | Overall Sample (N = 60) |
| --- | --- | --- | --- | --- | --- | --- |
|  | Group 1 (n = 12) | Group 2 (n = 12) | Group 3 (n = 12) | Group 4 (n = 12) | Group 5 (n = 12) |  |
| **Changes in behavior or personality** |  |  |  |  |  |  |
| Feeling frustrated | 4 (33.3) | 6 (50.0) | 3 (25.0) | 5 (41.7) | 3 (25.0) | 21 (35.0) |
| Depressed | 4 (33.3) | 1 (8.3) | 1 (8.3) | 3 (25.0) | 7 (58.3) | 16 (26.7) |
| Feeling anxious | 5 (41.7) | 1 (8.3) | 3 (25.0) | 2 (16.7) | 1 (8.3) | 12 (20.0) |
| Getting upset | 1 (8.3) | 1 (8.3) | 0 (0.0) | 4 (33.3) | 4 (33.3) | 10 (16.7) |

AD = Alzheimer’s disease

Note: Information was collected from patients only from Groups 1, 2, and 3; from care partners and patients (when able to self-report) in Group 4; and from care partners only in Group 5.

Note: Group-level data for the overall domain (changes in behavior or personality) is not provided as these symptoms were spontaneously reported and not assessed with all participants. Data shown are the number and percentage of individuals endorsing a symptom in each group and overall.

1. Current AD Symptoms Reported In Each Group and Overall: Concentration and Clear Thinking

| AD Symptom, n (%) | AD Classification | | | | | Overall Sample (N = 60) |
| --- | --- | --- | --- | --- | --- | --- |
|  | Group 1 (n = 12) | Group 2 (n = 12) | Group 3 (n = 12) | Group 4 (n = 12) | Group 5 (n = 12) |  |
| Concentration and clear thinking | 6 (50.0) | 6 (50.0) | 5 (41.7) | 12 (100.0) | 12 (100.0) | 41 (68.3) |
| Difficulty focusing or paying attention | 5 (41.7) | 6 (50.0) | 4 (33.3) | 10 (83.3) | 8 (66.7) | 33 (55.0) |
| Difficulty making decisions | 1 (8.3) | 2 (16.7) | 3 (25.0) | 9 (75.0) | 10 (83.3) | 25 (41.7) |
| Difficulty managing money; working with numbers | 1 (8.3) | 1 (8.3) | 1 (8.3) | 9 (75.0) | 12 (100.0) | 24 (40.0) |

AD = Alzheimer’s disease.

Note: Information was collected from patients only from Groups 1, 2, and 3; from care partners and patients (when able to self-report) in Group 4; and from care partners only in Group 5. Data shown are the number and percentage of individuals endorsing a symptom in each group and overall.

1. Current AD Symptoms Reported In Each Group and Overall: Orientation

| AD Symptom, n (%) | AD Classification | | | | | Overall Sample (N = 60) |
| --- | --- | --- | --- | --- | --- | --- |
|  | Group 1 (n = 12) | Group 2 (n = 12) | Group 3 (n = 12) | Group 4 (n = 12) | Group 5 (n = 12) |  |
| **Orientation** | 5 (41.7) | 4 (33.3) | 7 (58.3) | 10 (83.3) | 12 (100.0) | 38 (65.0) |
| Knowing the direction you need to go | 4 (33.3) | 3 (25.0) | 6 (50.0) | 9 (75.0) | 11 (91.7) | 33 (55.0) |
| Knowing the day, season, or time | 1 (8.3) | 2 (16.7) | 3 (25.0) | 7 (58.3) | 12 (100.0) | 25 (41.7) |
| Knowing where you are | 0 (0.0) | 1 (8.3) | 1 (8.3) | 7 (58.3) | 8 (66.7) | 17 (28.3) |
| Wandering^a^ | 0 (0.0) | 0 (0.0) | 1 (8.3) | 2 (16.7) | 2 (16.7) | 5 (8.3) |

AD = Alzheimer’s disease.

Note: Information was collected from patients only from Groups 1, 2, and 3; from care partners and patients (when able to self-report) in Group 4; and from care partners only in Group 5. Data shown are the number and percentage of individuals endorsing a symptom in each group and overall.

^a^ Wandering was not included in the list of symptoms presented to participants but was spontaneously reported by participants when discussing orientation-related symptoms.

1. Current AD Symptoms Reported In Each Group and Overall: Planning and Organizing

| AD Symptom, n (%) | AD Classification | | | | | Overall Sample (N = 60) |
| --- | --- | --- | --- | --- | --- | --- |
|  | Group 1 (n = 12) | Group 2 (n = 12) | Group 3 (n = 12) | Group 4 (n = 12) | Group 5 (n = 12) |  |
| Planning and organizing | 4 (33.3) | 4 (33.3) | 6 (50.0) | 11 (91.7) | 12 (100.0) | 37 (61.7) |
| Difficulty knowing what steps are next when performing a task | 2 (16.7) | 1 (8.3) | 4 (33.3) | 8 (66.7) | 11 (91.7) | 26 (43.3) |
| Difficulty understanding instructions to do something | 2 (16.7) | 2 (16.7) | 4 (33.3) | 8 (66.7) | 9 (75.0) | 25 (41.7) |
| Trouble planning events | 1 (8.3) | 1 (8.3) | 3 (25.0) | 7 (58.3) | 12 (100.0) | 24 (40.0) |

AD = Alzheimer’s disease.

Note: Information was collected from patients only from Groups 1,2, and 3; from care partners and patients (when able to self-report) in Group 4; and from care partners only in Group 5. Data shown are the number and percentage of individuals endorsing a symptom in each group and overall.

1. Current AD Symptoms Reported In Each Group and Overall: Dependence

| AD Symptom, n (%) | AD Classification | | | | | Overall Sample (N = 60) |
| --- | --- | --- | --- | --- | --- | --- |
|  | Group 1 (n = 12) | Group 2 (n = 12) | Group 3 (n = 12) | Group 4 (n = 12) | Group 5 (n = 12) |  |
| Remaining independent | 1 (8.3) | 2 (16.7) | 4 (33.3) | 11 (91.7) | 12 (100.0) | 30 (50.0) |
| Not being able to drive | 1 (8.3) | 2 (16.7) | 3 (25.0) | 10 (83.3) | 12 (100.0) | 28 (46.7) |
| Not being able to take care of yourself/needing to depend on others | 0 (0.0) | 0 (0.0) | 1 (8.3) | 7 (58.3) | 10 (83.3) | 18 (30.0) |
| Not making your own decisions about how you want to live your life | 0 (0.0) | 0 (0.0) | 1 (8.3) | 5 (41.7) | 10 (83.3) | 16 (26.7) |
| Having to move out of own home | 0 (0.0) | 0 (0.0) | 0 (0.0) | 4 (33.3) | 7 (58.3) | 11 (18.3) |
| Not being able to choose who helps you | 0 (0.0) | 0 (0.0) | 0 (0.0) | 2 (16.7) | 7 (58.3) | 9 (15.0) |
| Not able to choose where live or who with | 0 (0.0) | 0 (0.0) | 0 (0.0) | 2 (16.7) | 5 (41.7) | 7 (11.7) |

AD = Alzheimer’s disease.

Note: Information was collected from patients only from Groups 1-3, from care partners and patients (when able to self-report) in Group 4, and from care partners only in Group 5. Data shown are the number and percentage of individuals endorsing a symptom in each group and overall.
